# Integrative multiomics study for validation of mechanisms in radiation-induced ischemic heart disease in Mayak workers

**DOI:** 10.1371/journal.pone.0209626

**Published:** 2018-12-31

**Authors:** Anna Papiez, Omid Azimzadeh, Tamara Azizova, Maria Moseeva, Natasa Anastasov, Jan Smida, Soile Tapio, Joanna Polanska

**Affiliations:** 1 Institute of Automatic Control, Silesian University of Technology, ul. Akademicka 16, 44100 Gliwice, Poland; 2 Helmholtz Zentrum München-German Research Center for Environmental Health (GmbH), Institute of Radiation Biology, Ingolstädter Landstraße 1, 85764 Neuherberg, Germany; 3 Southern Urals Biophysics Institute, Ozyorsk, Russia; Northwestern University Feinberg School of Medicine, UNITED STATES

## Abstract

Previous studies have suggested that exposure to ionizing radiation increases the risk of ischemic heart disease (IHD). The data from the Mayak nuclear worker cohort have indicated enhanced risk for IHD incidence. The goal of this study was to elucidate molecular mechanisms of radiation-induced IHD by integrating proteomics data with a transcriptomics study on *post mortem* cardiac left ventricle samples from Mayak workers categorized in four radiation dose groups (0 Gy, < 100 mGy, 100–500 mGy, > 500 mGy). The proteomics data that were newly analysed here, originated from a label-free analysis of cardiac samples. The transcriptomics analysis was performed on a subset of these samples. Stepwise linear regression analyses were used to correct the age-dependent changes in protein expression, enabling the separation of proteins, the expression of which was dependent only on the radiation dose, age or both of these factors. Importantly, the majority of the proteins showed only dose-dependent expression changes. Hierarchical clustering of the proteome and transcriptome profiles confirmed the separation of control and high-dose samples. Restrictive (separate p-values) and integrative (combined p-value) approaches were used to investigate the enrichment of biological pathways. The integrative method proved superior in the validation of the key biological pathways found in the proteomics analysis, namely PPAR signalling, TCA cycle and glycolysis/gluconeogenesis. This study presents a novel, improved, and comprehensive statistical approach of analysing biological effects on a limited number of samples.

## Introduction

Mayak Production Association (MPA) is one of the biggest nuclear production facilities in Russia, housing plutonium production reactors and a reprocessing unit. Over decades, MPA has been running individual monitoring of the workers’ external radiation doses. These data have enabled the conduction of epidemiological studies that have shown a significant increase in ischemic heart disease incidence linearly correlated to the total external exposure [1–3], similar to other radiation-exposed populations [4, 5].

To elucidate biological mechanisms behind the increased heart disease risk, Azimzadeh et al. used a different approach [6]. A proteomic study was conducted using *post mortem* samples of cardiac left ventricle tissue from Mayak workers who were exposed to different external doses of ionizing radiation during their lifetime. Non-exposed individuals of the same area were used as the control population. Both controls and exposed workers died of ischemic heart disease [6]. This study clearly showed adverse dose-dependent alterations in the heart energy consumption that were strongly linked to the deactivation of peroxisome proliferator-activated receptor (PPAR) alpha, a key regulator of cardiac metabolism [7]. Furthermore, one of the main observations of the study was a dose-dependent increase in the number of downregulated mitochondrial proteins [6].

It is known from previous studies that, in ischemic conditions, the preferred energy source of the heart is glycolytic ATP derivation rather than the usage of fatty acid sources [8–11]. Moreover, ischemic heart conditions have been linked to alterations of the citric acid cycle [12]. However, in mild ischemic conditions, oxidation of fatty acids predominates over carbohydrate oxidation [13]. Interestingly, in the radiation-induced ischemic heart disease, a large number of proteins involved in glycolysis and fatty acid oxidation were down-regulated, indicating deactivation of both energy pathways [6], an exceptional situation in the cardiac energy paradigm [14].

The unique character of the collected heart material offers a wide range of possibilities to be explored using novel tools. This study aims to deepen further the knowledge of potential molecular mechanisms related to radiation-induced human heart pathology. It is firstly achieved by expanding the basic statistical analyses performed on raw proteomics data in [6], secondly by performing a gene expression analysis using whole-transcript cDNA sequencing (RNA-seq) methodology from a subset of the Mayak samples, and thirdly by integrating the newly generated proteomics and transcriptomics data sets. Using stepwise regression analysis, this study could separate the differentially regulated proteins in two categories: those that were deregulated only by the radiation dose and those that were dependent only on the age, which is an important development with regard to the study in [6]. Furthermore, the proteome-related mechanisms described previously could be partly confirmed by the transcriptome-linked processes.

## Materials and methods

### Proteomics samples

The cardiac left ventricle samples were derived *post mortem* from 29 radiation-exposed and non-exposed male individuals as described in detail in Azimzadeh et al. [6]. External radiation doses were estimated using the system “Dose-2005” [15]; individual annual doses from external gamma-ray doses were available for all workers in this study [16]. To avoid errors in the dose estimates, workers having cancer or other somatic diseases and thereby assumably additional non-occupational radiation exposure were excluded from this study [6]. These were classified into four groups according to the total dose of ionizing radiation to which the individuals were externally exposed: unexposed controls (3 samples), < 100 mGy low-dose exposed (6 samples), 100–500 mGy medium-dose exposed (10 samples) and > 500 mGy high-dose exposed (10 samples). The samples were subjected to an LC-MS/MS analysis as reported previously [6]. The metadata available for the samples included total external dose, age, smoking habits, alcohol consumption, and body mass index (BMI). Nonetheless, while factors such as dose and age varied among the individuals, all of the workers were recorded to be smokers and drinkers.

### RNA-seq samples

The RNA was derived from fresh frozen samples of 8 male individuals, a subset of the group previously analysed by a proteomics approach [6]. They were classified in two groups including unexposed controls (3 samples) and > 500 mGy high dose samples (5). The mirVana PARIS Kit (Ambion, ThermoFisher, USA) was used to isolate both native protein and total RNA. Total RNA was isolated from the lysate according to the manufacturer’s protocol (Ambion, ThermoFisher, USA). RNA integrity was assessed on the Agilent 2100 Bioanalyser. The transcriptomics analysis provided good quality data for four samples from the group of individuals used in the proteomics experiment: two controls (0008 and 0009) and two high-dose samples (0013 and 0014). These samples are suitable representatives of the control and high-dose groups, as none of them was identified as dose group outlier sample in [6]. The sequencing was performed using Illumina NextSeq 500 desktop sequencer.

## Informed consent

Samples used for the proteomics and RNA-seq experiments were collected post-mortem from donors who had previously given informed consent to participate in the study and who had consented to the processing of their personal data in accordance with the Russian Federal Laws No. 323-FL of 27.09.2013 and No. 261-FL of 25.07.2011. The study was approved by the Southern Urals Biophysics Institute’s Institutional Review Board.

### Statistical analysis of proteomics data

Outlier detection per each protein and endpoint was performed using Dixon’s criterion. The normality within dose groups was assessed using the Shapiro-Wilk [17] test. Based on the assumption notwithstanding, the Kruskal-Wallis test [18] with Storey’s False Discovery Rate (FDR) multiple testing threshold [19] was used as a non-parametric version of the ANOVA procedure for selecting differentiating proteins. FDR level of at most 5% was set as the selection criterion. As a post hoc method, the Dunnett test [20] was chosen for the identification of deregulated proteins among the dose groups with regard to the control samples. In the above test, the significance level was set to 0.05.

### Dose-age correlation analysis

For the proteomics data, the dose-age correlation was measured using Spearman’s rank correlation coefficient. Furthermore, to avoid a possible bias, regression analysis with backward stepwise model building was carried out. For protein levels in dose and age subgroups Box-Cox procedure was performed to transform the distribution to Gaussian [21]. Then, each protein expression level was modelled sequentially excluding factors, and model selection was performed using Akaike Information Criterion [22]. Following the modelling results, protein subgroups were identified as possible 1. non-dose related: in the final model only age related factor remained significant, with no significant impact of both dose factor and age-dose interaction (denoted as age-only dependent) 2. non-age related: in the final model only dose related factor remained significant, with no significant impact of both age factor and age-dose interaction (denoted as dose-only dependent).

### Functional analysis

Deregulated gene and protein enrichment of Gene Ontology (GO) Biological Process terms and Kyoto Encyclopedia of Genes and Genomes (KEGG) Pathways was assigned based on the results of Fisher’s exact test. Gene and protein interactions and signalling networks were analysed by the search tool STRING (http://string-db.org).

### Protein clustering

Clustering for protein samples was investigated using the hierarchical method with Spearman’s rank correlation as a similarity measure.

### Analysis of NGS data

The RNA-seq data were aligned and mapped using STAR software version 2.5.1 [23] against the GRCh38/hg38 human reference genome. Sorting and indexing were carried out with SAMtools version 1.3.1 [24]. Within group correlation analysis between biological replicates was carried out to assess the quality of the sequencing. Downstream differential expression analysis was assessed using the R DESeq2 package [25] with gene expression being modelled based on the negative binomial distribution.

### Integrative analysis

**Fig 1:**
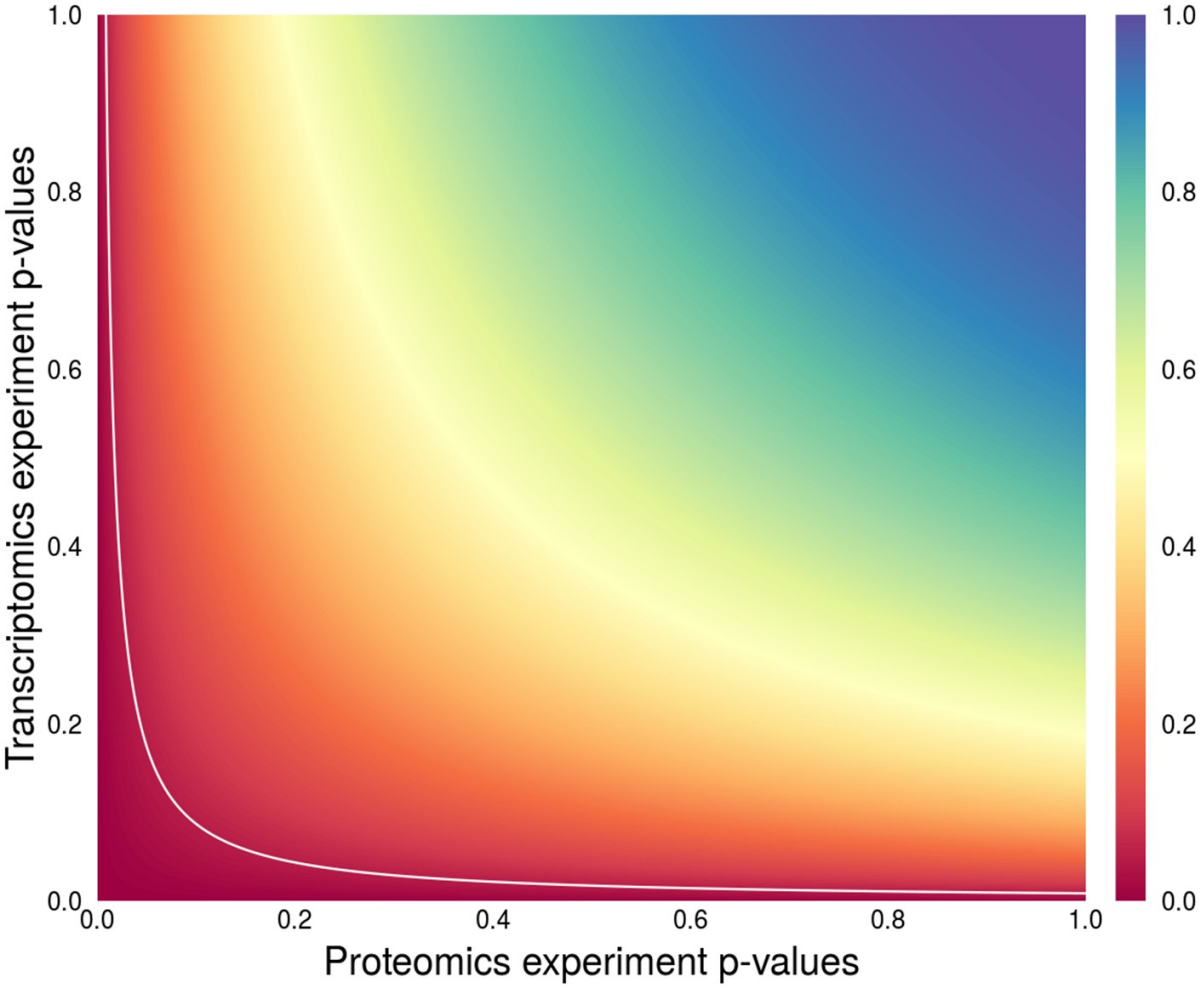
Illustration of the Fisher’s p-value combination method. The values on the axes represent p-values obtained in two individual experiments for matching features—in the case of this study proteins and transcripts. The colour depicts the combined p-value level. The white line illustrates the 0.05 threshold for the resulting combined p-value. The features with combined p-value below the white line are considered statistically significant.

For an integrative analysis of data from the proteomics and RNA-seq experiments, Fisher’s combined p-value transformation [26] was applied to the common gene and protein features (Fig 1). In the case of the proteomics data, the high-dose group of samples was compared to the control group to retain compatibility with the RNA-seq data. For proteins to which multiple genes corresponded, the gene with the minimum p-value was considered. The combined p-values were then corrected for multiple testing with the Benjamini-Hochberg method [27].

## Results and discussion

### Proteomics regression

The new analysis of the LC-MS/MS-based proteomics identified altogether 1, 281 proteins in the cardiac left ventricle samples. The outlier detection procedure for the MS data discovered no samples having a significant number of outlying protein abundance values.

**Fig 2:**
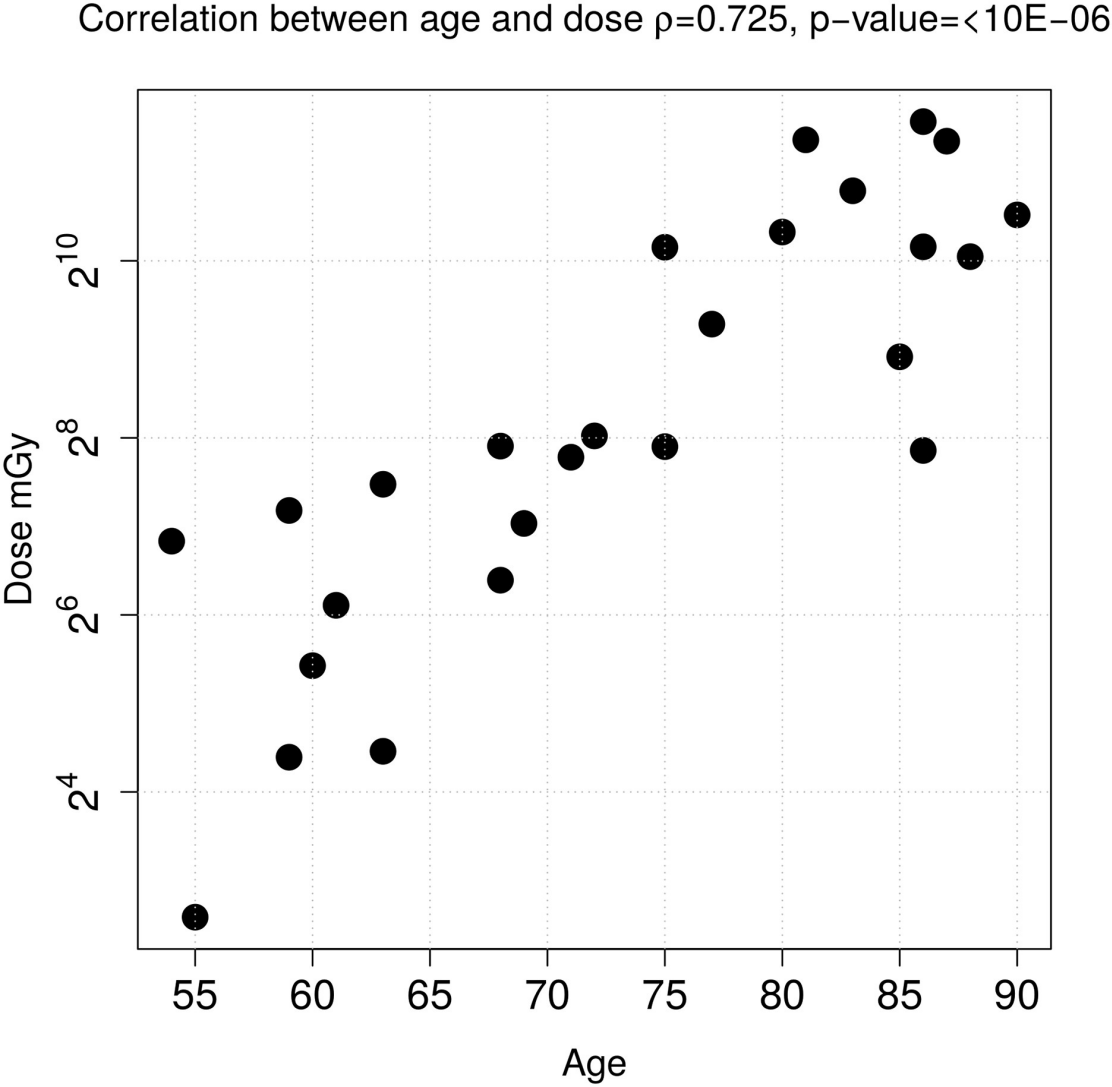
Scatter plot illustrating the data relationship between dose and age factors in the samples. Spearman’s correlation coefficient with a value of 0.725 is significant (p-value < 10*e* − 06).

The investigation indicated a strong positive correlation between the factors age and total external dose (Fig 2). However, no association was found between age or dose and BMI. The correlation test between BMI and age showed a p-value of 0.722 and between BMI and dose a p-value of 0.814. Therefore, multiple stepwise linear regression analyses with external dose and worker age as explanatory variables were conducted for each protein to identify those for which abundance variation was only dose dependent as opposed to proteins with age-only dependent variation or those for which none or both of the factors explained the existing variation. Additionally, BMI was also examined as a factor in the regression analysis. Smoking habits and alcohol consumption could not serve as explanatory variables due to the lack of variation among the studied individuals, all of whom were smokers and drinkers.

A total of 582 proteins (of 1, 281 identified) fell into the dose-only dependent (“dose-only”) category, 225 being only age-only dependent (“age-only”). In 212 cases the variation was best explained by a model containing both of these factors. For a total of only 17 proteins the BMI appeared as the explanatory variable. The complete list of proteins along with the factors that constitute their respective models is available in the S1 File.

Among dose-only dependent proteins, the most significantly related (p-value < 10 − 7) were: histidine ammonia lyase (HAL), zyg-11 family member A, cell cycle regulator (ZYG11A), RAD9-HUS1-RAD1 interacting nuclear orphan 1 (RHNO1), A-kinase anchoring protein 9 (LRG_331), moesin (MSN), acyl-CoA synthetase long chain family member 1 (ACSL1), isocitrate dehydrogenase 3 [NAD(+)] alpha (IDH3A), phosphoglycerate mutase 1 (PGAM1), chloride intracellular channel 1 (CLIC1), malic enzyme 1 (ME1), glycogen phosphorylase (PYGM), aladin WD repeat nucleoporin (AAAS), and ribosomal protein S27a (RPS27A). Many of these proteins are directly involved in the energy metabolism.

Impact of the age factor was in general weaker. Only three proteins, radixin (RDX), coatomer protein complex subunit beta 2 (COPB2), and protein arginine methyltransferase 5 (PRMT5) showed significance at p-value < 10 − 5 in age-only models.

The dose-only and age-only dependent proteins were examined using Gene Ontology (GO) and Kyoto Encyclopedia of Genes and Genomes (KEGG) pathway enrichment analyses. The most significant pathways according to GO terms were “oxidation-reduction process” and “respiratory electron transport chain” for dose-only and age-only deregulated proteins, respectively. The most significant KEGG pathways were “metabolic pathways” and “oxidative phosphorylation” for dose-only and age-only deregulated proteins, respectively. The complete lists of age- and dose-only dependent overrepresented GO and KEGG terms are available in the S2 File.

Some heart-relevant enriched age-only, dose-only, and overlapping KEGG pathways are also presented in Table 1. Despite the existing strong correlation between dose and age factors in the proteomics data, a majority of the deregulated proteins was demonstrated to be dose-only dependent. The comparison of the pathways activated by both age-only dependent and dose-only dependent proteins emphasised general heart pathologies such as Hypertrophic cardiomyopathy and Dilated cardiomyopathy or processes linked to energy metabolism (Metabolic pathways, Propanoate metabolism, Oxidative phosphorylation). However, regarding dose-only related pathways, the findings confirmed formerly reported metabolic networks [6], including PPAR signalling, Glycolysis, Fatty acid metabolism, and TCA cycle.

**Table 1:** KEGG pathways enriched by proteins that were identified as dose-only dependent and/or age-only dependent in the backward stepwise regression model selection procedure. The pathways common for dose-only and age-only dependent proteins are placed in the middle.

Age-dependent	Dose-dependent	
Fatty acid elongation	PI3K-Akt signalling pathway	Ribosome
Tryptophan metabolism	Pathogenic Escherichia coli infection	Carbon metabolism
	Protein processing in endoplasmic reticulum	Glyoxylate and dicarboxylate metabolism
	Biosynthesis of amino acids	Arrhythmogenic right ventricular cardiomyopathy
	Proteasome	Pyruvate metabolism
**Dose-age-dependent**	Tight junction	Butanoate metabolism
Metabolic pathways	Glycolysis/Gluconeogenesis	Adrenergic signalling in cardiomyocytes
Cardiac muscle contraction	Peroxisome	AMPK signaling pathway
Propanoate metabolism	Leukocyte transendothelial migration	Vasopressin-regulated water reabsorption
Valine, leucine and isoleucine degradation	Fatty acid metabolism	Beta-Alanine metabolism
Hypertrophic cardiomyopathy	ECM-receptor interaction	Antigen processing and presentation
Dilated cardiomyopathy	PPAR signalling pathway	Phagosome
Oxidative phosphorylation	Fatty acid degradation	TCA cycle
	Porphyrin and chlorophyll metabolism	2-Oxocarboxylic acid metabolism
	Focal adhesion	

### ∪–shape and ∩–shape protein filtration analysis

**Fig 3:**
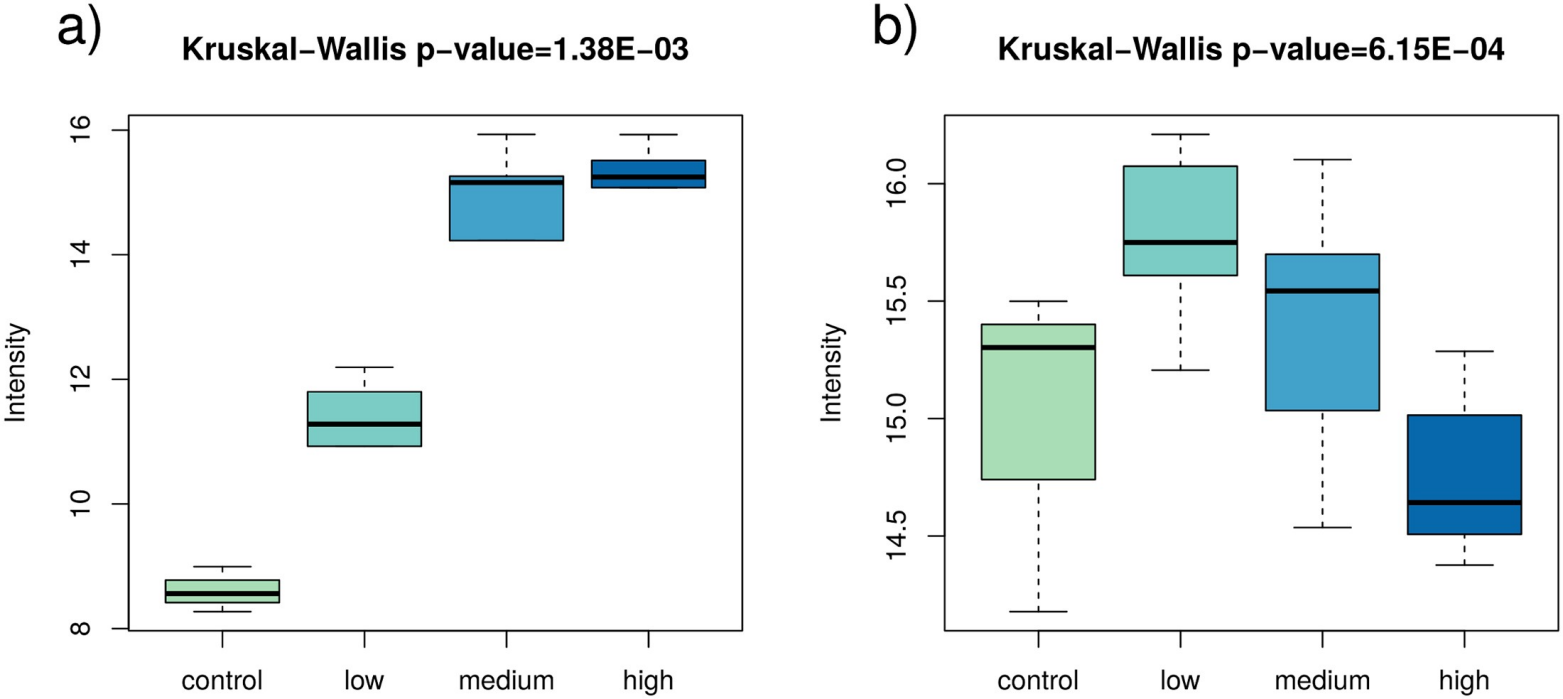
Example boxplots for two proteins identified in the regression analysis as dose-only dependent. The left plot represents a situation where the protein level gradually grows (Kruskal-Wallis p-value 1.38E-03), whereas the right the protein level forms a ∩–shape with an increase in the low-dose groups and a decrease in the higher doses (Kruskal-Wallis p-value 6.15E-04).

Even though the regression analysis revealed proteins the expression profile of which changed with the dose, it did not further clarify situations where changes showed no linear dependence of the dose. This situation may be denoted as a ∪–shape or a ∩–shape. The former occurs when the protein level is relatively high in the control group, then decreases with dose, and then increases again towards higher doses. The latter is unequivocal with the situation where the protein level is low in the control group, increases with dose, and at the highest doses declines. It is illustrated in Fig 3.

To analyse such cases and obtain a clear picture of the existing differentiation with filtered out ∪–shape or a ∩–shape proteins, tests for protein deregulation with regard to the control samples were carried out on the proteomics data [6]. Subgroups of low- (< 100 mGy), medium- (100–500 mGy) and high-dose (> 500 mGy) exposed individuals were all compared to the non-exposed control group. The Lilliefors test showed that the normality assumption was not fulfilled in the subgroups. Therefore, the Kruskal-Wallis test for differentiation between the dose groups was performed. It resulted in 582 proteins significantly deregulated between at least one of the four dose groups (control, < 100 mGy, 100–500 mGy, and > 500 mGy), which is consistent with the results of the regression analysis (582 dose-only dependent proteins). The post-hoc Dunnett tests allowed to identify sets of deregulated proteins specific for each external dose group (Table 2). Although the expression of many proteins increased with the dose, an overwhelming majority of all differentially regulated proteins were identified as down-regulated.

**Table 2:** Numbers of dose-only dependent deregulated proteins in different dose groups in comparison to the non-exposed controls resulting from post-hoc Dunnett tests.

With reference to controls	< 100 mGy	100–500 mGy	>500 mGy
**Up-regulated proteins**	1	15	12
**Down-regulated proteins**	2	33	307
**∪–shape and ∩–shape proteins**	260		

**Fig 4:**
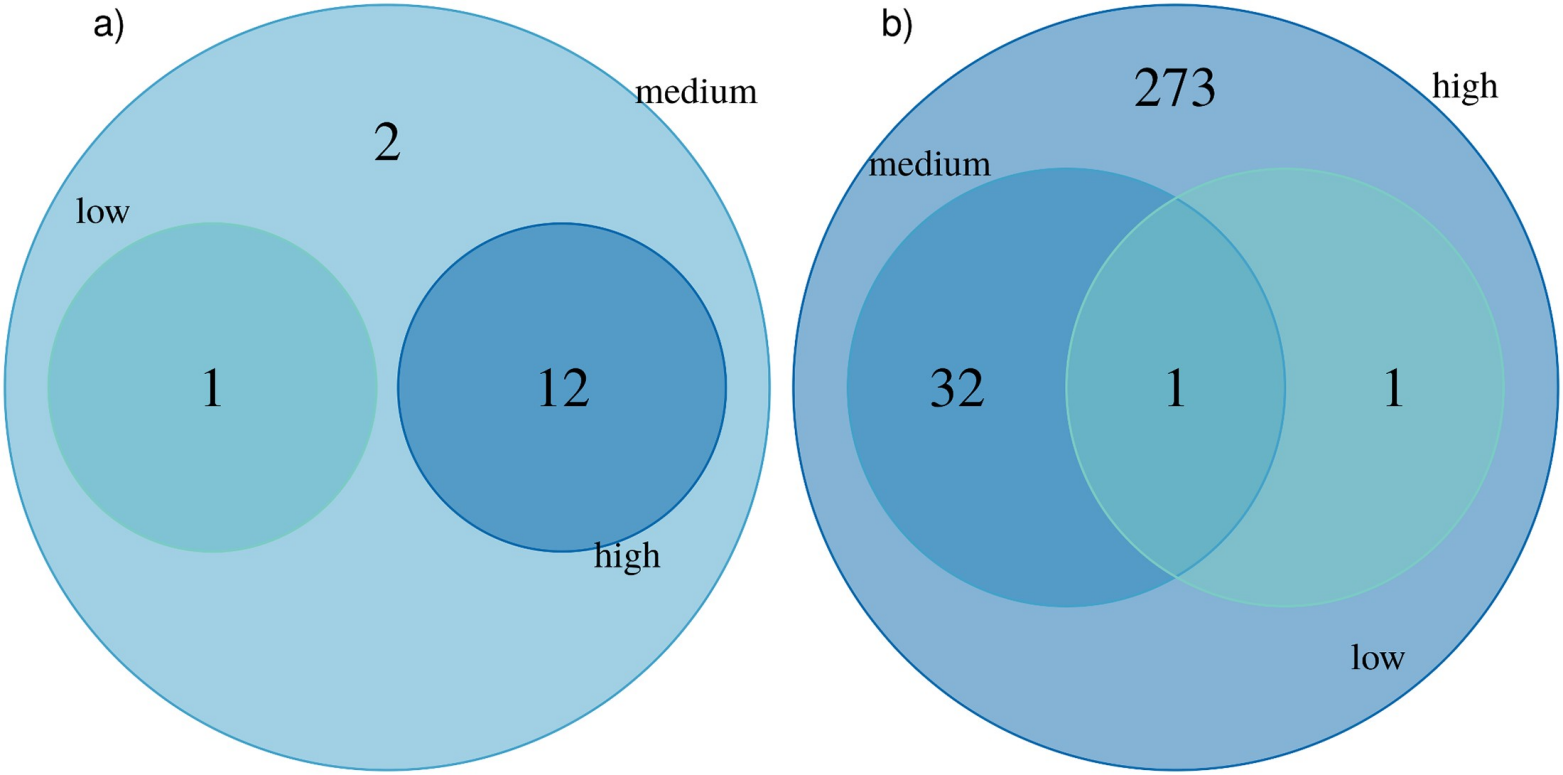
Venn diagrams presenting the numbers and overlap of significantly a) up-regulated and b) down-regulated proteins in different dose groups among dose-only dependent proteins with respect to the control according to Dunnett’s test at *α* = 0.05.

The numbers of total and shared strictly up- or down-regulated proteins in different dose groups for dose-only dependent proteins are presented using Venn diagrams in Fig 4.

In the group of up-regulated proteins, 12 proteins were significantly changed when compared to unexposed samples in both high and medium dose groups. Only one uncharacterized protein (C1orf112) was common for up-regulated proteins in low and medium dose individuals, but its abundance among samples of the high-dose group did not differ significantly from the unexposed samples. None of the proteins was significantly up-regulated in all dose groups. Most of these up-regulated proteins were present in both medium- and high-dose exposed individuals, suggesting that their expression has a dominant dose-dependent nature.

When down-regulated proteins were analysed, the cytochrome c oxidase assembly factor (COX20) protein demonstrated significant deregulation in all dose groups in relation to the control samples. Therefore, it may represent a switch-type biomarker of radiation exposure. This protein is essential in the assembly of cytochrome C oxidase, an important component of the respiratory pathway.

Additionally, there were 32 common differentially regulated proteins between the medium and high doses (ALAD, ARHGAP11A, ATP5L, BFSP1, C14orf2, CA2, CCDC141, CRAT, DLD, EIF2B5, FECH, FNDC3A, HSD17B4, ITGA6, LAP3, LGALS3BP, LRG_391, LRRC37B, MCCC1, MEMO1, MLYCD, MYOM3, NDUFB11, OLA1, OTUB1, PCBD2, RAB5A, RXRA, SLC25A3, SUCLA2, UCHL3, WIPI1). Several of these proteins are located in the mitochondria (12) and/or have metabolic functions. Most of the down-regulated proteins (273) appear only in the high dose group, which suggests that their expression is not linearly dose-dependent, but rather by a high dose threshold.

### Analysis of differentially regulated transcripts

In the RNA-seq analysis, 25,221 transcripts were identified within the 4-sample data set representing two control and two high-dose samples. The biological replicates within the control and high-dose groups showed a high correlation of *ρ* = 0.976 between the two control samples and *ρ* = 0.940 between the two high dose group samples. The DEseq adaptive threshold method led to discarding transcripts with less than 15 counts mapped from further analysis. The negative binomial test with Benjamini-Hochberg multiple testing correction resulted in comparable numbers of 979 significantly up-regulated transcripts and 895 significantly down-regulated transcripts.

### Proteomics and transcriptomics data integration

As the proteomics data were to be merged with the RNA-seq data, only the high-dose exposed samples could be taken into further consideration in the integration process. The full lists of differentially expressed genes and deregulated proteins along with the corresponding p-values are compiled in the S3 File.

**Fig 5:**
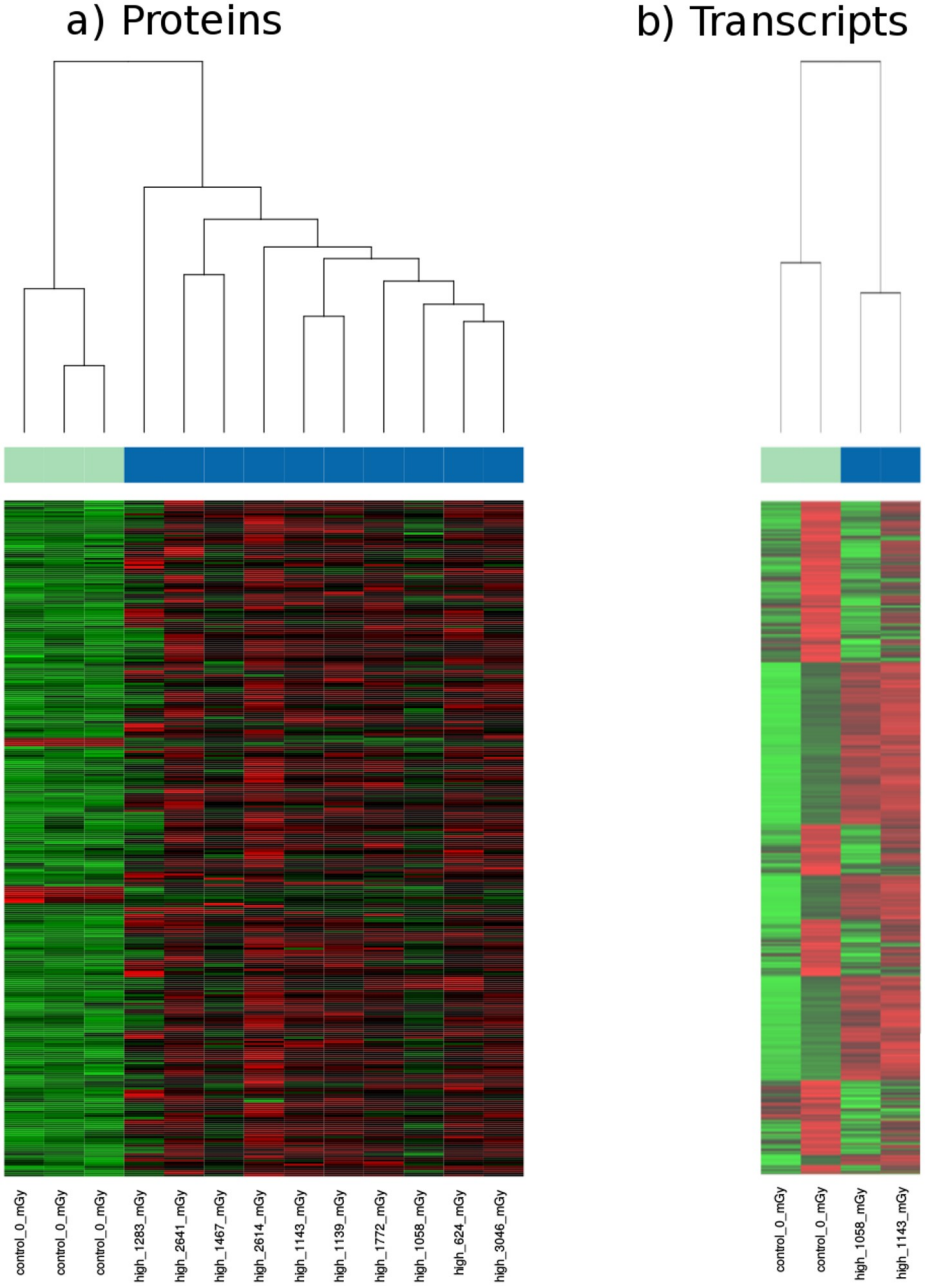
Supervised heat map showing the separation of high-dose samples from controls based on a) 319 dose-dependent significantly deregulated proteins; and b) 1,874 significantly deregulated transcripts. The numbers provided next to the class label show the total external dose of an individual. The colour bars indicate sample groups: cyan—controls, blue—high-dose samples.

When applying hierarchical clustering in the proteome, the dose-only proteins showed a clear separation between high-dose samples and controls (Fig 5). By comparison, the supervised heat map analysis using the age-only related proteins did not cluster the individuals by age in a consistent manner but still rather by their respective doses S1 Fig. Similarly, in the transcriptome, the two high-dose samples could be separated from the controls as seen in Fig 5.

Taking into account only the common deregulated proteins and transcripts at the significance level of 5% in the two data sets, further defined as the restrictive approach, only 2 proteins (ANK3, P4HTM) remained statistically significantly up-regulated and 30 down-regulated (ACADM, ANXA1, ANXA5, CALM2, CAP1, CD93, DCN, DLD, DPT, DSTN, EIF4A2, ERAP1, GLRX, GRPEL1, HNRNPK, HSPA8, ITGA6, LAP3, LGALS1, LUM, NIPSNAP3A, NIPSNAP3B, PDIA3, RAB5A, RBBP7, RPS4X, RPS6, SDPR, SUCLG1, UBE2N).

**Fig 6:**
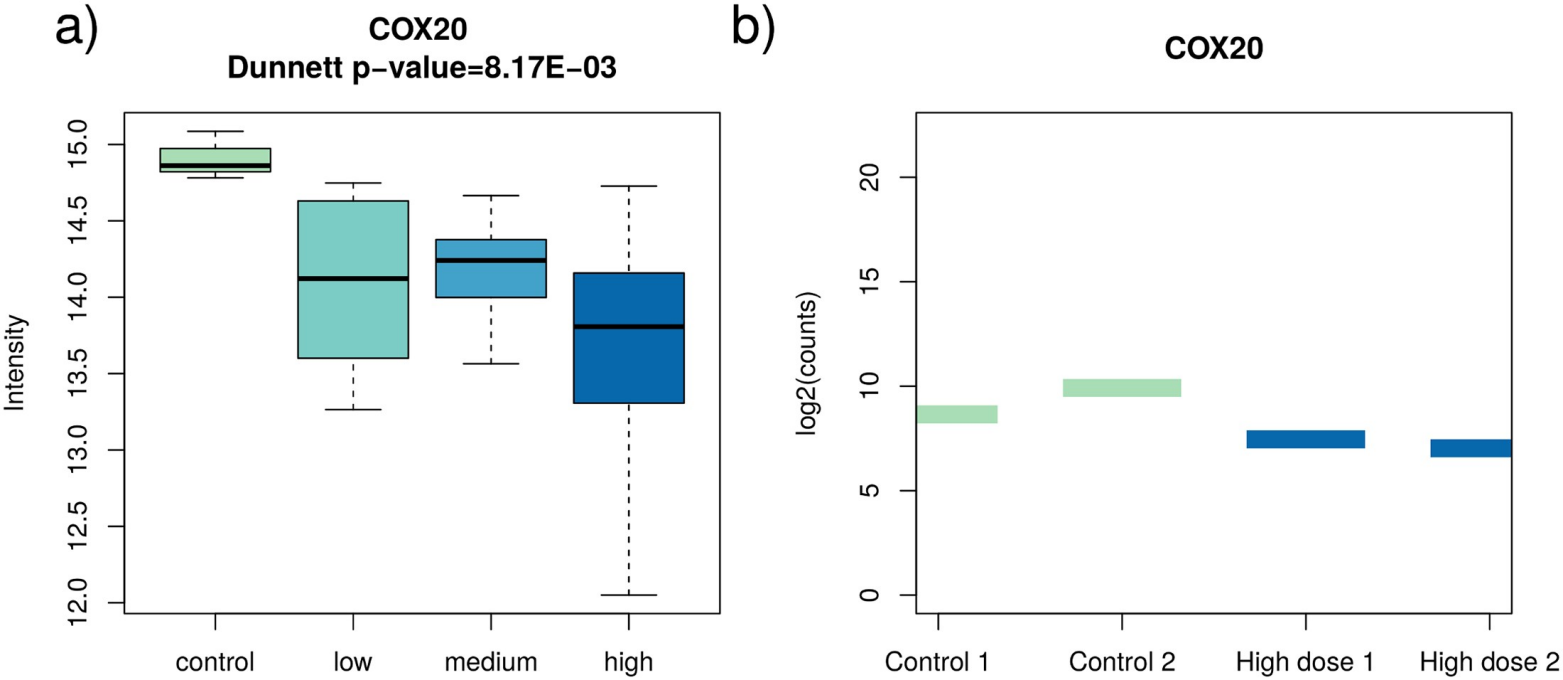
The down-regulated COX20 shown as an exemplary coherent deregulated transcript-protein pair. The boxplots in **a)** show the statistical summary of protein expression values in each dose group, whereas the bars in plot **b)** present the gene expression for each available individual transcript sample on the logarithmic scale. The plot illustrates the declining direction of protein expression, and though there is a large range of values in the high-dose group distribution, a significant difference is observed compared to the control group (Dunnett test p-value 8.17E-03). Likewise, in the RNA-seq data gene expression drops in this case.

The full list of transcript/protein pairs identified using the integrative approach is collected in S4 File. All of the considered transcript/protein pairs were coherent regarding the direction of the deregulation, as exemplarily shown in Fig 6. Several of the down-regulated gene/protein pairs were members of the pathways RNA binding, Oxidoreductase activity, and Poly(A) RNA binding (Table 3). The lists of KEGG pathways enriched by deregulated transcript/protein pairs identified using integrative and restrictive analysis approaches are presented in S5 File.

**Table 3:** KEGG signalling pathways overrepresented by gene-protein pairs found to be significantly deregulated in high-dose samples in comparison to controls. The pathways in the left column were obtained from the intersection of enriched pathways from significant genes and proteins in the two data sets. The pathways in the right column were enriched by gene-protein features significant by the combined Fisher’s p-value method.

Restrictive approach	Integrative approach	
Proteasome	Glycolysis / Gluconeogenesis	Beta-Alanine metabolism
Ribosome	Oxidative phosphorylation	Metabolic pathways
Proteoglycans in cancer	Citrate cycle (TCA cycle)	Tryptophan metabolism
Pathogenic Escherichia coli infection	Bacterial invasion of epithelial cells	Arginine and proline metabolism
**Propanoate metabolism**		Lysine degradation
	Phagosome	PPAR signalling pathway
	Vasopressin-regulated water reabsorption	Proximal tubule bicarbonate reclamation
	Ascorbate and aldarate metabolism	Terpenoid backbone biosynthesis
	Valine, leucine and isoleucine degradation	Glyoxylate and dicarboxylate metabolism
	Histidine metabolism	Fatty acid degradation
	Pyruvate metabolism	Carbon metabolism

Among the overrepresented GO terms, there were 241 Biological Process ontologies found with the restrictive approach and 54 derived from the integrative approach (see Materials and methods/ Integrative analysis). Twenty-four of these enriched terms were common between the two methods. The full list of overrepresented ontologies is available in S6 File.

All in all, the integrative approach proved supreme over the restrictive comparison in the validation of the proteomics data by the transcriptomics analysis. The significance in terms of Fisher’s combined p-value gene-protein pairs connected to enriched KEGG pathways accentuated radiation-linked processes such as PPAR signalling, TCA cycle and Glycolysis/Gluconeogenesis whilst the intersection of KEGG terms overrepresented in the separately analysed proteomics and transcriptomics data were scarce and general in nature, mentioning, for instance, Proteasome, or Ribosome. However, these represent two main cellular machineries highly dependent on energy supply for cellular functions and include proteins important in oxidoreductase activity (Proteasome) and RNA binding proteins (Ribosome).

### Study limitations

This study has limitations. It includes only male workers and the number of samples, especially in the transcriptomics study, is very low. Collecting the samples *post mortem* is naturally a limiting factor that, however, cannot be changed in future studies. However, state-of-the-art tools for sequencing data analysis, i.e. DESeq render possible the processing of even such small sample sizes. Possible uncertainties in the individual doses, especially in the first decade of operation at MPA, 1948–1958, cannot be excluded.

## Conclusion

The substantial contribution of this study compared to the previous proteomics analysis [6] was the use of custom statistical methods to distinguish dose-only dependent protein expression changes from the age-only dependent changes. By the use of an integrative statistical analysis approach, adapted to the nature of the analysed data, the discovered proteomic processes could be verified by the gene expression study. Pathways such as glycolysis, oxidative phosphorylation, citric acid cycle and, importantly, PPAR signalling were confirmed using a novel p-value integration technique, as opposed to the use of a conventional restrictive result comparison procedure. This non-standard pipeline for merging data sets opens the door for new statistical methodologies and emphasises the importance of careful planning in the stepwise analysis in order to obtain valid conclusions.

## Supporting information

S1 Fig Heatmap showing hierarchical clustering with the use of age-only dependent deregulated proteins. The heatmap labels show the dose group and the age of an individual in years. It illustrates that the samples still cluster according to dose, rather than age, while using significantly deregulated age-only dependent features. The colour bars indicate sample groups: cyan—controls, blue—high-dose samples.
(TIF)

S1 File Dose-only and age-only dependent proteins with the regression coefficient p-values. (XLSX)

S2 File Gene Ontology biological Process terms and KEGG pathways enriched with dose-only and age-only dependent proteins. (XLSX)

S3 File Genes and proteins deregulated by high radiation dose. (XLSX)

S4 File Integrative approach deregulated transcript-protein pairs. (XLSX)

S5 File KEGG pathways enriched by deregulated transcript/protein pairs identified using integrative and restrictive analysis approaches. (XLSX)

S6 File Gene Ontology biological process terms enriched with significantly deregulated proteins identified with the restrictive and integrative approach. (XLSX)
