# Supplementary figures and images for: Integrative multiomics study for validation of mechanisms in radiation-induced ischemic heart disease in Mayak workers
Source: PLoS One. 2018 Dec 31;13(12):e0209626. doi: 10.1371/journal.pone.0209626 (PMC6312255; doi:10.1371/journal.pone.0209626)

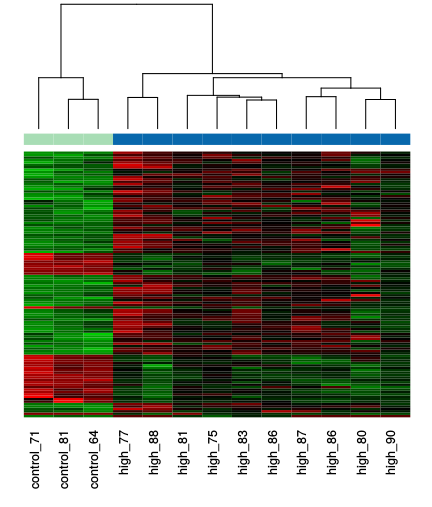

Supplement: S1 Fig — The heatmap labels show the dose group and the age of an individual in years. It illustrates that the samples still cluster according to dose, rather than age, while using significantly deregulated age-only dependent features. The colour bars indicate sample groups: cyan—controls, blue—high-dose samples. (TIF) [file pone.0209626.s001.tif]
